# Early Extubation After Pediatric Liver Transplantation: From Baseline Vulnerability to Perioperative Readiness

**DOI:** 10.3390/children13101346

**Published:** 2026-10-03

**Authors:** Devan Kowdley, Gennaro Martucci, Valeria Sottani, Gaetano Burgio, Daniela Damian

**Affiliations:** 1 School of Medicine, University of Pittsburgh, Pittsburgh, PA 15261, USA; dkowdley@mgb.org; 2 Department of Anesthesiology, Perioperative and Pain Medicine, Brigham and Women’s Hospital, Boston, MA 02115, USA; 3 Department of Anesthesia and Intensive Care, IRCCS-ISMETT (Istituto Mediterraneo per i Trapianti e Terapie ad Alta Specializzazione), 90127 Palermo, Italy; gmartucci@ismett.edu (G.M.); gburgio@ismett.edu (G.B.); 4 Department of Anesthesia and Intensive Care, Faculty of Medicine, University of Palermo, 90127 Palermo, Italy; vsottani@ismett.edu; 5 Department of Anesthesiology and Perioperative Medicine, School of Medicine, University of Pittsburgh, Pittsburgh, PA 15261, USA; 6 Department of Anesthesiology and Perioperative Medicine, UPMC Children’s Hospital of Pittsburgh, 4401 Penn Ave, Pittsburgh, PA 15224, USA

**Keywords:** early extubation, pediatric liver transplantation, enhanced recovery, transfusion, hemodynamics, analgesia, graft function

## Abstract

**Highlights:**

**Main findings**

- Extubation readiness is a dynamic perioperative state integrating baseline vulnerability, intraoperative physiological recovery, and early postoperative validation.
- Immediate or early extubation is feasible in carefully selected children and is associated with shorter intensive-care and hospital stays, although current retrospective evidence does not establish causality.

**Implications of the main findings**

- Age, body weight, and disease-severity scores should inform risk assessment rather than serve as absolute exclusions; multidisciplinary end-of-case assessment should guide extubation decisions.
- Standardized definitions and prospective multicenter validation are needed before universal readiness criteria or independent outcome benefits can be established.

**Abstract:**

Early extubation after pediatric liver transplantation (PLTx) is increasingly incorporated into enhanced-recovery pathways, yet definitions, selection criteria, and outcome reporting remain inconsistent. This narrative review synthesizes evidence identified through PubMed/MEDLINE (January 2000 through August 2026), citation screening, and supplementary screening of recent reports. Immediate extubation denotes tracheal extubation in the operating room, whereas early extubation has been defined through 24 postoperative hours; delayed extubation and prolonged mechanical ventilation are similarly heterogeneous. The available evidence informs a dynamic framework in which preoperative characteristics define baseline vulnerability, the intraoperative course reflects whether that vulnerability has been overcome or amplified, and the early postoperative course validates the decision. Young age, low body weight, growth failure, or higher disease-severity scores may increase risk but should not function as absolute exclusions; conversely, low scores may underestimate disease-specific risk in metabolic disorders. Direct readiness indicators include effective ventilation, neurological and quantitative neuromuscular recovery, normothermia, stable hemodynamics without escalating vasoactive support, controlled bleeding, satisfactory abdominal closure, and improving acid–base status and lactate clearance. Early extubation is associated with shorter intensive-care and hospital stays in selected cohorts, but causality remains uncertain because evidence is predominantly retrospective and single-center. Standardized definitions and prospective multicenter validation are required.

## 1. Introduction

Extubation after pediatric liver transplantation (PLTx) has evolved from a postoperative milestone into a potential intraoperative endpoint. Postoperative mechanical ventilation was historically routine, whereas contemporary enhanced-recovery practice increasingly regards immediate or early extubation as the culmination of coordinated anesthetic, surgical, hemodynamic, and metabolic recovery. The decision remains consequential: continued ventilation may prolong sedative exposure, increase ventilator-related morbidity, and consume intensive-care resources, whereas premature extubation carries the risk of urgent reintubation. Extubation timing should therefore be viewed not as an isolated end-of-case decision, but as an integrated marker of perioperative homeostasis.

Early extubation after liver transplantation was first explored in adult recipients, beginning with a report linking fluid restriction to early extubation and followed in 1997 by a dedicated two-center experience with immediate operating-room extubation and a separate single-center cohort supporting the feasibility of early tracheal extubation [1,2,3]. The first dedicated pediatric report identified in this review appeared in 2003, and subsequent single-center studies established the feasibility of immediate extubation in selected children [4,5,6].

During the past decade, the pediatric literature has expanded considerably, paralleling the adoption of protocolized perioperative care and the development of dedicated multidisciplinary transplant teams. Establishment of a specialized pediatric liver-transplant anesthesia team has been associated with higher rates of early extubation and fewer postoperative ventilator days, while recent cohorts have documented progressive increases in immediate and early extubation over time [7,8]. Collectively, these findings suggest that successful implementation depends not only on patient selection, but also on institutional experience and coordinated anesthetic, surgical, and postoperative management.

Despite this progress, terminology remains inconsistent. Immediate extubation generally refers to removal of the endotracheal tube in the operating room, whereas early extubation has been defined using postoperative thresholds ranging from 3 to 24 h. Definitions of delayed extubation and prolonged mechanical ventilation are similarly heterogeneous, ranging from ventilation beyond 24–48 h to several postoperative days [9,10,11,12]. This variability complicates direct comparison of extubation rates, reintubation, complications, and resource utilization across centers.

Previous pediatric publications have largely described single-center feasibility, extubation rates, or individual associated factors. This review extends that literature by integrating baseline patient vulnerability, the intraoperative physiological trajectory, directly observed end-of-case readiness, and early postoperative graft and surgical evolution into an evidence-informed multidisciplinary framework. Extubation readiness is therefore considered not solely an anesthetic endpoint but the product of patient condition, operative course, and early post-transplant recovery.

This narrative review aims to clarify current terminology, summarize the reported incidence of immediate and early extubation after PLTx, and synthesize the preoperative, intraoperative, and early postoperative factors associated with successful or delayed ventilator liberation.

## 2. Materials and Methods

A structured narrative search of PubMed/MEDLINE was conducted for English-language publications from January 2000 and finalized on 27 August 2026. The principal search combined (“liver transplantation”[MeSH Terms] OR “liver transplant*”[Title/Abstract]) AND (“child”[MeSH Terms] OR “infant”[MeSH Terms] OR “adolescent”[MeSH Terms] OR pediatric[Title/Abstract] OR paediatric[Title/Abstract] OR child*[Title/Abstract] OR infant*[Title/Abstract] OR adolescent*[Title/Abstract]) AND (“airway extubation”[MeSH Terms] OR extubat*[Title/Abstract] OR “immediate extubation”[Title/Abstract] OR “early extubation”[Title/Abstract] OR “mechanical ventilation”[Title/Abstract] OR “ventilator liberation”[Title/Abstract] OR reintubat*[Title/Abstract] OR “delayed extubation”[Title/Abstract] OR “prolonged ventilation”[Title/Abstract]). Predictor-related terms were not required to preserve sensitivity. Reference lists and forward citations were screened manually, and eligible recent reports not yet indexed in PubMed/MEDLINE were also considered.

Two reviewers independently screened titles and abstracts and subsequently assessed potentially eligible full-text articles under the oversight of two senior authors. Disagreements were resolved through discussion and consensus. Eligible studies included patients younger than 18 years undergoing liver transplantation and addressed the timing, feasibility, safety, associated factors, or outcomes of immediate or early extubation, delayed extubation, extubation failure, reintubation, or prolonged postoperative ventilation. Adult-only studies were excluded from the pediatric evidence synthesis, although selected seminal adult studies were retained for historical context and enhanced-recovery principles.

Contemporary pediatric studies directly evaluating immediate or early extubation are summarized in Table 1. Studies of delayed extubation, extubation failure, and prolonged mechanical ventilation complemented the analysis of unsuccessful or delayed ventilator liberation. Additional perioperative studies and guidelines were included when they addressed anesthetic, hemodynamic, transfusion, surgical, respiratory, nutritional, or metabolic factors relevant to extubation readiness.

**Figure 1:**
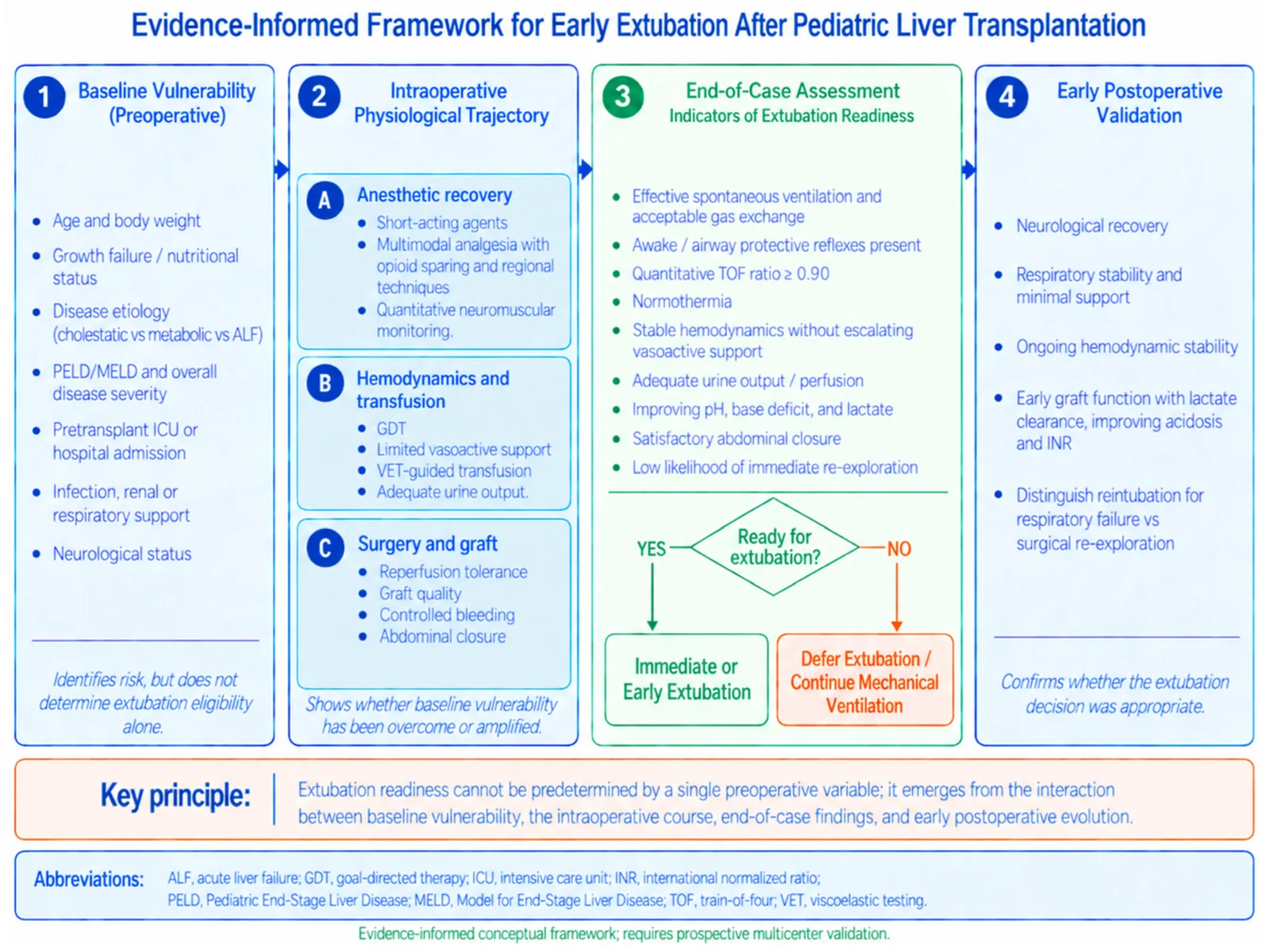
Evidence-informed clinical framework for early extubation after pediatric liver transplantation. Baseline vulnerability informs risk; the intraoperative trajectory and end-of-case assessment support a multidisciplinary extubation decision; and early postoperative evolution validates the decision. The framework is conceptual and is not a validated score or universal checklist.

Study-specific definitions were retained throughout extraction. Conflicting temporal thresholds were not retrospectively harmonized; instead, findings were grouped narratively according to each study’s original definition, and rates derived from non-equivalent categories were not pooled. Because of substantial clinical and methodological heterogeneity, no meta-analysis or formal risk-of-bias assessment was performed. OpenAI ChatGPT was used for translation, language editing, manuscript organization, and bibliographic formatting, and the OpenAI image-generation tool within ChatGPT was used in developing Figure 1. No generative-AI tool was used to determine study eligibility, extract study data, or independently interpret the evidence. The authors verified all sources, citations, interpretations, and final wording.

## 3. Definitions, Reported Incidence, and Extubation Failure

### 3.1. Definitions

Despite increasing adoption, the pediatric liver-transplant literature lacks a standardized temporal definition of early extubation. Immediate extubation most consistently refers to removal of the endotracheal tube in the operating room, whereas early extubation has included thresholds of 3 h or less, on-table or within 4 h, within 24 h, and by postoperative day 1 [8,9,11,12,13]. Study-specific definitions and reported rates are summarized in Table 1.

Definitions at later time points are also heterogeneous. Delayed extubation has been defined as extubation beyond 24 h, after postoperative day 1, or at or beyond 48 h [8,10,13,14]. Prolonged mechanical ventilation is not synonymous with delayed extubation and has been defined using thresholds from more than 24 h to at least four postoperative days [15,16].

For this narrative synthesis, early extubation is used as an umbrella term encompassing immediate operating-room extubation and early postoperative extubation within 24 h. Extubation beyond 24 h is described as delayed unless an original study used a different threshold, and prolonged mechanical ventilation is retained only as defined by the investigators. Non-equivalent categories were not pooled or treated as directly comparable.

**Table 1:** Contemporary studies evaluating immediate or early extubation after pediatric liver transplantation (2018–2026).

Study	Design and Population	Sample Size	Extubation Definition and Rate	Key Findings
**Gurnaney et al., 2018** [17]	Single-center retrospective cohort; Children’s Hospital of Philadelphia; pediatric LT, July 2011–December 2014.	64	Immediate/early extubation: extubation in the OR. Achieved in 37/64 (57.8%); successful extubation required no reintubation within 24 h.	Immediately extubated patients were older, more often admitted from home, less often listed as UNOS status 1A, and received less RBC and FFP. None required reintubation within 24 h. Immediate extubation was associated with shorter PICU stay (3.8 vs. 17.6 days) and hospital stay (10.7 vs. 29.7 days).
**Ünlükaplan et al., 2020** [18]	Single-center retrospective cohort; Başkent University; pediatric orthotopic LT, April 1996–August 2009.	57	Immediate extubation: extubation in the OR/no postoperative mechanical ventilation. Immediate extubation: 31/57 (54%); postoperative ventilation: 26/57 (46%).	Growth failure (OR 37) and higher intraoperative lactate (OR 1.5) were independently associated with postoperative ventilation. Ventilated patients also had greater transfusion and vasopressor requirements.
**Sahinturk et al., 2021** [19]	Single-center retrospective cohort; Başkent University; pediatric LT, January 2012–December 2017.	81	Immediate extubation: extubation in the OR. Achieved in 39/81 (48%); 42/81 were extubated in the ICU.	Non-immediate extubation was associated with massive hemorrhage, greater RBC transfusion, higher intraoperative lactate, vasopressor use, and open abdomen. Immediate extubation was associated with fewer infections, shorter ICU stay (2.0 vs. 4.5 days), and lower hospital mortality (0% vs. 12%).
**Yoeli et al., 2022** [20]	Single-center retrospective cohort; Children’s Hospital Colorado; isolated primary LT, January 2015–December 2020.	81	Immediate extubation: extubation in the OR. Achieved in 58/81 (72%).	Age <2 years, preoperative mechanical ventilation, and higher total intraoperative epinephrine and dopamine requirements were independently associated with lower odds of immediate extubation. Immediate extubation was associated with shorter ICU and hospital stays; model AUC 0.89.
**Pandey et al., 2022** [21]	Single-center retrospective historical before-and-after cohort; living-donor LT in children ≤ 20 kg; nonprotocol era 2014–2016 versus protocol era 2017–2019.	134 (58 nonprotocol; 76 protocol)	Immediate extubation: extubation in the OR. Rate increased from 48.3% to 85.5% after protocol implementation.	Reintubation decreased from 10.9% to 4.6%. The protocol era had shorter hospital stay and lower mortality. High vasopressor support was the only factor independently associated with non-immediate extubation during the protocol era; temporal comparisons remain susceptible to cointerventions.
**Saeyup et al., 2023** [9]	Single-center retrospective cohort; Thailand; pediatric living-donor LT, January 2013–December 2021.	79	Immediate extubation: OR extubation, 19/79 (24%). Early extubation: within 24 h, 12/79 (15%). Combined immediate/early group: 31/79 (39%).	Shorter anhepatic time was the only factor independently associated with immediate/early extubation. The immediate/early group had shorter ICU stay. Delayed patients had more total and early medical complications and more reintubations; hospital stay did not differ significantly.
**Sahin et al., 2023** [22]	Single-center retrospective cohort; pediatric LT.	64	Immediate extubation: extubation in the OR. Achieved in 45/64 (70.3%); 19/64 were extubated in the PICU.	Delayed patients received more FFP and platelets and had longer anesthesia, PICU stay, and hospital stay. Reintubation occurred in 2/45 (4.4%) immediate versus 3/19 (15.8%) delayed patients.
**Baba et al., 2024** [13]	Single-center retrospective evaluation of center-specific selection criteria and anesthetic protocol; Japan; pediatric LT, January 2016–December 2020.	266 cases	Immediate extubation: extubation in the OR, 81/266 (30.5%). Among non-immediate cases, early extubation was by POD1; delayed extubation was after POD1.	All 81 immediately extubated cases remained extubated without respiratory-failure reintubation. Among 185 non-immediate cases, 130 were extubated by POD1. Seven of 53 delayed cases required tracheostomy, identifying inability to extubate by POD1 as a high-risk marker.
**Scalise et al., 2024** [23]	Single-center retrospective cohort; Boston Children’s Hospital; pediatric LT, January 2005–November 2022.	235	Immediate extubation: extubation in the OR. Achieved in 164/235 (69.8%); 2 immediate-extubation patients were reintubated within 3 days.	Pretransplant ICU care, higher PELD/MELD, greater transfusion and pressor requirements, and longer cold ischemia time were associated with lower odds of immediate extubation; age and weight were not. Immediate extubation was associated with shorter ICU and hospital stays and increased from approximately 40% to 83% over time.
**Goldstein et al., 2024** [24]	Single-center retrospective multidisciplinary-pathway cohort; Children’s Healthcare of Atlanta; pediatric LT, 2015–2021.	173	Immediate extubation: extubation in the OR. Achieved in 121/173 (69.9%); reintubation occurred in approximately 2.5%.	Immediately extubated patients were older and had lower PELD/MELD, less transfusion, shorter surgery, and more frequent primary abdominal closure. Immediate extubation was associated with less HFNC use, fewer infections, shorter PICU stay, and lower mortality.
**Gokula et al., 2025** [11]	Single-center retrospective observational cohort; pediatric living-donor LT; age 3 months–10 years, January 2022–June 2024.	28	Early extubation: on-table or ≤4 h postoperatively. Twenty-three patients (82.1%) were extubated on-table; 5 (17.9%) were ventilated > 4 h.	Early extubation was feasible across the studied age range. Interpretation is limited by the small, selected cohort. The report supports safety and feasibility more strongly than definitive predictor identification.
**Yang and Zhang, 2026** [12]	Single-center retrospective cohort; Beijing Friendship Hospital; pediatric LT, September 2019–November 2022.	256	Early extubation: ≤3 h after surgery. Achieved in 155/256 (61%).	Higher preoperative albumin and greater intraoperative urine output were associated with a higher likelihood of early extubation. Higher WBC, respiratory infection, higher end-of-surgery lactate, and DCD graft were associated with a lower likelihood. Urine output had the strongest discrimination (AUC 0.898). ICU stay was slightly shorter, without differences in 30-day complications or hospital stay.
**Martucci et al., 2026** [25]	Single-center retrospective cohort; IRCCS-ISMETT, Palermo; consecutive pediatric PLTx, January 2018–July 2023; 75% living-related grafts.	99	Immediate extubation: planned extubation at the end of surgery when predefined clinical criteria were met. Achieved in 89/99 (90%).	Immediate extubation was associated with no vasopressor requirement, shorter surgical time, and lower end-of-operation lactate. Thirty-four percent received no PRBC; 77% received <20 mL/kg; plasma or platelets were given to 17 children. The study supports feasibility of transfusion-sparing management and IE within an experienced multidisciplinary program.
**Fahmy et al., 2026** [8]	Single-center retrospective cohort; high-volume North American center; isolated primary LT, January 2013–December 2021.	245	Immediate extubation: intraoperative/OR, 33/245 (13.5%). Early extubation: ≤24 h, 110/245 (44.9%). Delayed extubation: >24 h, 102/245 (41.6%).	No reintubations occurred in the immediate/early groups. Immediate/early extubation was associated with shorter ICU and hospital stays. Recipient age, preoperative care setting, and regional anesthesia were independently associated with immediate/early extubation; model C-statistic 0.82.

Abbreviations: AUC, area under the receiver operating characteristic curve; DCD, donation after circulatory death; FFP, fresh frozen plasma; HFNC, high-flow nasal cannula; ICU, intensive-care unit; LT, liver transplantation; MELD, Model for End-Stage Liver Disease; OR, operating room (or odds ratio when followed by a numerical effect estimate); PELD, Pediatric End-Stage Liver Disease; PICU, pediatric intensive-care unit; POD1, postoperative day 1; RBC, red blood cell; UNOS, United Network for Organ Sharing; WBC, white blood cell. Definitions of immediate and early extubation varied among studies and are reproduced as reported by the investigators. Note: All studies were retrospective and single-center. Reported extubation rates are not directly comparable between centers because study populations, graft types, institutional pathways, temporal definitions, and reintubation assessment windows differed substantially. Associations between extubation timing and outcomes are susceptible to selection bias and confounding by preoperative illness severity and intraoperative stability. The Pandey study used a historical before-and-after design. The Scalise cohort updates the earlier Boston Children’s Hospital single-center experience.

### 3.2. Reported Incidence and Extubation Failure

Reported rates of immediate extubation vary markedly among contemporary pediatric cohorts, ranging from 13.5% to 90% [8,25]. This range should not be interpreted as a direct comparison of institutional performance, because the studies differ in recipient age and illness severity, graft type, study era, selection criteria, perioperative protocols, and the definition applied. For example, a recent cohort of 99 consecutive recipients reported immediate extubation in 89/99 (90%), whereas a larger North American cohort reported immediate extubation in 13.5% and early postoperative extubation within 24 h in an additional 44.9% [8,25]. Reported incidence must therefore be interpreted in the context of each study population and temporal definition rather than pooled as a single summary rate.

Successful extubation is also defined inconsistently. Some studies required the absence of reintubation within 24 h, whereas others assessed reintubation over longer intervals or reported it without a clearly specified observation window [17,23,24]. Reintubation was uncommon in most selected immediate- and early-extubation cohorts, but the reported rates are not directly comparable because the denominator, follow-up interval, and reasons for reintubation differed among studies.

A dedicated study of extubation failure reported a substantially higher failure rate of 21.1%; however, it evaluated children who arrived in the intensive-care unit intubated and were subsequently extubated rather than children selected for immediate operating-room extubation. Muscle fatigue was the most frequent reason for reintubation, while other causes included hemodynamic instability, upper-airway obstruction, inadequate respiratory drive, and return to the operating room [26]. This population and endpoint should therefore not be compared directly with immediate-extubation cohorts.

Future studies should report the exact location and timing of extubation, the duration and type of postextubation respiratory support, the observation window for reintubation, and the clinical reason for reintubation. Reintubation for airway or respiratory failure should also be distinguished from reintubation required for surgical re-exploration. Standardized reporting of these elements is necessary for meaningful comparison of extubation success and safety among pediatric transplant centers.

## 4. Preoperative Patient-Related Factors: Baseline Vulnerability

Factors associated with extubation timing differ in their temporal proximity to the final decision. Preoperative characteristics represent relatively distal indicators of risk: they identify children with potentially limited respiratory, nutritional, renal, neurological, or metabolic reserve but rarely determine eligibility for immediate or early extubation in isolation. Their principal value is to anticipate perioperative complexity and define the intensity of reassessment required at the completion of transplantation.

### 4.1. Age, Body Weight, Growth Failure, and Disease Etiology

The indications for pediatric liver transplantation vary with age. Biliary atresia and other cholestatic disorders predominate during infancy, whereas metabolic and genetic diseases account for a larger proportion of transplantations in older children. Infants constitute a distinct perioperative population and may require greater resource utilization, particularly at very young ages or extremely low body weights [27]. These observations, however, do not establish age or weight as direct determinants of extubation readiness.

Although immediate extubation was historically reserved for older and larger recipients, contemporary studies demonstrate that it can be achieved across a broad range of ages and body sizes. In a cohort of 235 pediatric recipients, immediate extubation was achieved in 69.8% of cases, without an independent association between extubation status and recipient age or weight [23]. Another cohort included recipients as young as four months and found no significant demographic differences between children extubated in the operating room and those who remained intubated [19]. Similarly, protocolized perioperative management increased immediate-extubation rates among children weighing 20 kg or less; although age and weight were associated with extubation status on univariable analysis, only high vasopressor support remained independently associated with non-immediate extubation during the protocolized period [21].

Nutritional status may be more informative than body size alone. Growth failure is common in children with end-stage liver disease and has been independently associated with postoperative mechanical ventilation [18]. Preoperative albumin, although influenced by hepatic synthetic function, inflammation, and fluid status, may provide an indirect measure of nutritional and physiological reserve. In one cohort, higher preoperative albumin was independently associated with early extubation, with an ROC-derived cutoff of 36.15 g/L or greater; however, discrimination was modest (AUC 0.663), and this threshold should be considered study-specific rather than universal [12].

Objective assessment of muscle mass may provide information not captured by weight, growth failure, or serum albumin alone. In a retrospective cohort of 276 pediatric liver-transplant recipients, computed tomography-derived psoas muscle area was lower in children with end-stage liver disease than in those with acute liver failure and showed weak associations with one-year survival, hospitalization duration, and PELD score [28]. Although extubation timing was not evaluated, reduced muscle mass may identify diminished physiological reserve relevant to postoperative respiratory recovery.

Disease etiology is relevant primarily because it shapes the child’s associated vulnerabilities rather than because it independently determines extubation timing. Cholestatic disease may be accompanied by malnutrition, portal hypertension, and major transfusion requirements, whereas metabolic disorders may present with preserved hepatic synthetic function but substantial biochemical or neurological risk. Age, weight, and diagnosis should therefore be interpreted as markers of anticipated complexity rather than absolute contraindications to immediate or early extubation.

### 4.2. Disease Severity, Pretransplant Clinical Status, and Risk Scores

The Pediatric End-Stage Liver Disease (PELD) score was developed to estimate wait-list mortality and guide organ allocation rather than to assess cardiopulmonary reserve, respiratory muscle function, or perioperative recovery. Its ability to predict immediate extubation is therefore inherently limited.

Associations between PELD and extubation timing have been inconsistent. Some cohorts found no significant difference in PELD scores between children extubated in the operating room and those who remained intubated [6,19]. In contrast, larger recent studies identified higher PELD or Model for End-Stage Liver Disease (MELD) scores as unfavorable factors for immediate extubation, although substantial proportions of recipients with greater disease severity were still successfully extubated in the operating room [23,24]. PELD may therefore contribute to preoperative risk stratification but should not be used as an independent exclusion criterion.

Conversely, a low calculated PELD score may be falsely reassuring in children undergoing transplantation for liver-based metabolic disorders. In urea-cycle defects, hepatic synthetic function may remain preserved, and transplantation is performed to prevent recurrent hyperammonemic crises and irreversible neurological injury rather than to treat decompensated end-stage liver disease. A pediatric series reported a mean calculated PELD score of −0.5 at transplantation, illustrating the limited ability of conventional allocation scores to reflect disease-specific metabolic risk and the need for exception-based prioritization in these patients [29,30].

Extubation readiness in metabolic disease should therefore incorporate diagnosis-specific biochemical and neurological endpoints rather than PELD alone. A Japanese series of liver transplantation for ornithine transcarbamylase deficiency reported postoperative hyperammonemia in recipients of grafts from heterozygous carrier donors and described a recipient extubated on postoperative day 5 [31]. Extubation timing was not a formal outcome of that study; nevertheless, the experience illustrates why immediate extubation may appropriately be deferred when sustained ammonia control, neurological recovery, or effective metabolic correction by the graft remains uncertain. Subsequent experience from the same center emphasized that selection for immediate extubation should account for disease-specific physiological characteristics [13].

Clinical indicators of reduced physiological reserve may provide more specific information than PELD alone. Pretransplant hospitalization, cirrhosis, age younger than one year, and the need for continuous renal replacement therapy were independently associated with prolonged mechanical ventilation and were incorporated into the PROVE-ALT score [32]. Preoperative respiratory infection, elevated white blood cell count, and lower albumin have also been associated with a reduced likelihood of early ventilator liberation [12].

Preoperative assessment should therefore extend beyond a single disease-severity score. Age, nutritional status, pretransplant location, renal or respiratory support, active infection, neurological status, and disease phenotype collectively describe baseline vulnerability. These factors estimate risk but do not determine the final extubation decision. Whether baseline vulnerability persists at the completion of transplantation depends principally on the intraoperative physiological trajectory.

## 5. Intraoperative Factors Associated with Extubation Readiness: Physiological Trajectory

Intraoperative factors are temporally closer to the extubation decision and are more modifiable than baseline characteristics. They indicate whether the physiological stress of transplantation has been adequately controlled and whether baseline vulnerability has translated into persistent hemodynamic, respiratory, hematological, or metabolic instability.

### 5.1. Anesthetic Management and Neuromuscular Recovery

Readiness for immediate extubation requires predictable anesthetic recovery without compromising hemodynamic stability or graft perfusion. End-stage liver disease, altered protein binding, major fluid shifts, and the anhepatic phase may alter drug distribution and clearance; short-acting agents with limited dependence on hepatic metabolism are therefore generally favored and should be titrated to the evolving physiological condition [21,33].

No single anesthetic technique has been established as superior. Contemporary strategies commonly combine a volatile or intravenous hypnotic with short-acting opioids and multimodal non-opioid analgesia, aiming to prevent residual sedation and respiratory depression rather than to eliminate any individual drug class.

Opioid-sparing techniques may facilitate recovery of consciousness and spontaneous ventilation. Regional techniques, including fascial-plane blocks, may be combined with acetaminophen, dexmedetomidine, and other non-opioid adjuncts in selected recipients, with careful consideration of thrombocytopenia and coagulopathy [34]. Regional anesthesia was independently associated with immediate or early extubation in one retrospective cohort; this observation does not establish a causal effect and may reflect patient selection and broader pathway adherence [8].

Quantitative neuromuscular monitoring is essential because clinical signs may fail to identify residual blockade after prolonged surgery, hypothermia, metabolic disturbance, or organ dysfunction. A train-of-four ratio of at least 0.90 should be documented before extubation. Rocuronium with sugammadex or organ-independent agents such as cisatracurium may permit predictable recovery when appropriately monitored and titrated [35].

Anesthetic and neuromuscular recovery are necessary but not sufficient conditions for immediate extubation. An awake child with complete reversal should remain intubated when hemodynamic, surgical, respiratory, or metabolic homeostasis has not been restored.

### 5.2. Hemodynamic Stability, Perfusion, Fluid Management, and Transfusion

Persistent hemodynamic instability is one of the most consistent reasons to defer immediate extubation. Children who remained mechanically ventilated after transplantation had higher lactate concentrations, greater vasoactive and transfusion requirements, and more frequent major hemorrhage in several cohorts [18,19].

Higher intraoperative epinephrine and dopamine requirements were independently associated with lower odds of operating-room extubation, and high vasopressor support remained the principal factor associated with non-immediate extubation after pathway implementation [20,21]. Greater pressor and transfusion requirements were similarly associated with non-immediate extubation in other large cohorts [23]. Conversely, greater urine output and lower end-of-surgery lactate were associated with extubation within three hours [12]. In a recent 99-recipient cohort, immediate extubation was associated with no vasopressor requirement, shorter surgery, and lower end-of-operation lactate [25]. These variables should be interpreted as markers of intraoperative severity and recovery rather than established causes.

Fluid management should preserve graft and end-organ perfusion while avoiding unnecessary interstitial and pulmonary edema. Restriction should not be equated with under-resuscitation: infants and small children have limited circulating-volume reserve and may be vulnerable to hypotension from excessive restriction, whereas positive fluid balance may worsen tissue edema, respiratory mechanics, and graft injury [36,37,38]. Individualized goal-directed therapy (GDT) should integrate arterial pressure, perfusion, dynamic hemodynamic assessment, urine output, lactate trajectory, blood loss, and surgical phase rather than rely on a fixed volume target. In selected recipients, perioperative echocardiography—including transesophageal echocardiography when feasible and supported by appropriate expertise—may complement GDT by identifying otherwise unexplained instability and guiding fluid and blood-product administration, particularly in children with underlying cardiac disease [39].

Hemostatic management should avoid both empiric overtransfusion and delayed correction of clinically important bleeding. Viscoelastic testing (VET) can characterize the changing coagulation profile and support targeted component replacement [40]. In the recent transfusion-sparing cohort, 34% of recipients required no packed red blood cells, 77% received less than 20 mL/kg, and plasma or platelets were administered to only 17 of 99 children [25]. These data support the feasibility of targeted blood-product administration within a coordinated program but do not establish reduced transfusion as a cause of successful extubation. Transfusion requirements should instead be interpreted principally as markers of hemorrhage, surgical complexity, and physiological instability.

Persistent hypotension, escalating vasoactive support, rising or non-clearing lactate, inadequate urine output, ongoing hemorrhage, uncontrolled coagulopathy, or continued major transfusion at abdominal closure indicate incomplete restoration of homeostasis and should prompt deferral of immediate extubation.

### 5.3. Surgical and Graft Considerations

Surgical and graft-related variables influence extubation readiness through blood loss, ischemia–reperfusion physiology, graft function, abdominal mechanics, and the likelihood of early reintervention. Total operative duration alone has not been consistently associated with extubation success because it does not adequately describe the physiological severity of the procedure.

Dedicated pediatric liver-transplant teams may improve the consistency of intraoperative management and the ability to recognize extubation readiness. Establishment of a specialized pediatric liver-transplant anesthesia team was associated with more early extubations and fewer postoperative ventilator days, while coordinated surgical and anesthesia expertise may facilitate lower transfusion requirements and more standardized decision-making [7,39].

The relationship between graft type and extubation timing is inconsistent. Recipients of whole grafts were more frequently extubated in the operating room in one cohort, while deceased-donor partial grafts were associated with lower odds of immediate extubation on univariable analysis; however, this association did not persist after multivariable adjustment and appeared to be influenced by recipient age and vasopressor requirements [20]. Receipt of a deceased-donor or donation-after-circulatory-death graft was independently associated with a lower likelihood of extubation within three hours in another study [12]. Other cohorts demonstrated that immediate extubation could be achieved across graft categories [23].

Living-donor-only cohorts have reported high immediate-extubation rates, particularly following implementation of protocolized pathways [11,21]. These studies do not include deceased-donor comparators and therefore cannot establish living donation as an independent predictor. The apparent influence of graft category may instead be mediated by graft quality, graft-to-recipient size, ischemic time, recipient age, blood loss, hemodynamic response to reperfusion, and the feasibility of primary abdominal closure.

Graft type should therefore not function as an isolated eligibility criterion. The clinically relevant question is whether the graft and surgical course have permitted restoration of hemodynamic and metabolic stability without excessive bleeding, respiratory compromise from abdominal closure, or a high likelihood of immediate surgical re-exploration.

### 5.4. Integrated End-of-Case Assessment: Indicators of Extubation Readiness

At the completion of transplantation, directly observed physiological indicators should carry greater weight than baseline risk factors. The criteria below constitute an evidence-informed clinical synthesis rather than a prospectively validated score or universal checklist.

The child should demonstrate effective spontaneous ventilation and acceptable gas exchange without excessive ventilatory pressures; adequate neurological recovery with airway-protective reflexes; quantitative neuromuscular recovery with a train-of-four ratio of at least 0.90; normothermia; stable hemodynamics without escalating vasoactive support; satisfactory perfusion and urine output; controlled bleeding without an ongoing major transfusion requirement; and abdominal closure without clinically important respiratory or hemodynamic compromise. Evidence of early graft function should include improvement rather than deterioration in pH, base deficit, and lactate. A recent high-extubation-rate cohort operationalized immediate extubation using end-of-surgery hemodynamic stability, temperature above 36 °C, adequate neurological responsiveness, satisfactory graft Doppler assessment, and lactate clearance [25], closely aligning with this multidomain approach.

Individual studies have proposed specific end-of-surgery thresholds, including pH 7.30 or greater or lactate 3.55 mmol/L or less, but these values have not been prospectively validated as universal pediatric criteria and should not replace assessment of the overall trajectory [12,23]. Similarly, increased driving pressure may identify impaired respiratory-system compliance and a higher likelihood of delayed extubation, but it should be interpreted together with oxygenation, ventilation, abdominal mechanics, and the remainder of the clinical examination [14].

This conceptual framework requires prospective multicenter validation before use as a formal decision tool.

A practical clinical framework for extubation readiness after pediatric liver transplantation is shown in Figure 1.

## 6. Immediate Postoperative Validation of the Extubation Decision

Immediate or early extubation is not the final endpoint of perioperative recovery. The early postoperative course determines whether respiratory autonomy, neurological recovery, hemodynamic stability, and graft function persist after transfer from the operating room. Postoperative monitoring therefore validates the extubation decision rather than simply documenting that the endotracheal tube has been removed.

### 6.1. Respiratory and Neurological Stability

Successful early ventilator liberation requires sustained spontaneous ventilation, adequate gas exchange, stable respiratory effort, and preservation of airway-protective reflexes. Progressive tachypnea, increasing oxygen or noninvasive respiratory-support requirements, hypercapnia, reduced cough, upper-airway obstruction, or signs of respiratory muscle fatigue may indicate that physiological reserve was overestimated.

Neurological recovery should include an appropriate level of consciousness, effective cough and swallowing, and the absence of progressive encephalopathy or residual sedative effect. These criteria are particularly important in children with pre-existing neurological injury or metabolic disease, in whom normal hepatic synthetic function or a low PELD score may not reflect neurological readiness.

Normothermia should also be maintained after transfer. Persistent hypothermia may delay drug metabolism, impair coagulation, trigger shivering, and increase oxygen consumption, thereby destabilizing an otherwise marginal respiratory state. Early postoperative evaluation should therefore integrate temperature, neurological function, respiratory effort, oxygenation, ventilation, and the trajectory of respiratory-support requirements.

A substantially higher extubation-failure rate has been reported among children who arrived in the intensive-care unit intubated and underwent later extubation than among selected operating-room-extubation cohorts. Respiratory muscle fatigue, hemodynamic instability, upper-airway obstruction, inadequate respiratory drive, and the need for surgical reintervention were among the reported causes [26]. These findings reinforce the importance of distinguishing primary respiratory or airway failure from reintubation performed for surgical re-exploration.

### 6.2. Metabolic and Graft-Function Trajectory

Improving metabolic homeostasis at the end of transplantation supports extubation readiness, but continued improvement after transfer provides stronger evidence of effective graft function. Serial lactate, pH, base deficit, glucose, coagulation parameters, urine output, and hemodynamic trends are more informative than any isolated value.

Lactate clearance is particularly relevant because persistent hyperlactatemia may reflect impaired graft function, inadequate perfusion, ongoing bleeding, catecholamine exposure, or a combination of these factors. A decreasing lactate accompanied by correction of acidosis and stable hemodynamics supports the appropriateness of early extubation, whereas rising lactate or recurrent acidosis should prompt urgent evaluation for circulatory, graft-related, or surgical complications.

Respiratory or hemodynamic deterioration after initial extubation may also reflect graft-related or surgical complications rather than primary respiratory failure. In a retrospective imaging series of 119 pediatric liver-transplant recipients, hepatic artery and portal-vein complications and ischemic infarction were more often identified early, whereas biliary strictures and some venous or parenchymal complications emerged later [41]. Although that study did not evaluate extubation, it supports prompt assessment for vascular, biliary, parenchymal, and extraparenchymal complications when respiratory, hemodynamic, or metabolic recovery deviates from the expected trajectory.

Later postoperative biomarkers may provide important prognostic information but should not be confused with criteria for immediate extubation. For example, the INR × lactate product measured at 72 postoperative hours has demonstrated strong discrimination for early mortality, but it was developed as a prognostic indicator rather than an extubation-readiness measure [42]. Its principal relevance to this review is that the trajectory of metabolic and synthetic recovery continues beyond the immediate extubation window.

Successful immediate or early extubation and satisfactory graft recovery are therefore related but not interchangeable outcomes. The absence of respiratory failure does not exclude early graft dysfunction, and delayed graft recovery does not invariably require reintubation. Postoperative validation should remain multidimensional, integrating respiratory, neurological, hemodynamic, surgical, and metabolic evolution.

## 7. Discussion

The central synthesis of this review is that extubation readiness after pediatric liver transplantation is not a fixed preoperative characteristic but an evolving perioperative state. Preoperative characteristics define baseline vulnerability, the intraoperative course reflects whether that vulnerability has been overcome or amplified, and the end-of-case examination provides the most direct evidence of readiness. The early postoperative course then validates whether respiratory autonomy and systemic homeostasis have been sustained. This framework helps reconcile inconsistent associated factors across studies: young age, low body weight, growth failure, elevated PELD/MELD scores, pretransplant hospitalization, and organ dysfunction may reduce the likelihood of immediate extubation but do not determine the outcome independently [6,12,18,23,32].

The limitations of static preoperative prediction are particularly evident at both extremes of the PELD spectrum. A high score may identify severe liver disease and greater anticipated perioperative complexity without necessarily precluding operating-room extubation. Conversely, a low calculated score in a child with a liver-based metabolic disorder may underestimate biochemical and neurological vulnerability because hepatic synthetic function can remain relatively preserved. Preoperative scores should therefore inform preparation and risk stratification rather than establish an irreversible decision regarding postoperative ventilation. The final determination should remain responsive to the child’s hemodynamic, respiratory, surgical, neurological, and metabolic trajectory during transplantation.

Early extubation should be interpreted as both a therapeutic objective and a marker of uncomplicated perioperative recovery. Across multiple cohorts, early-extubated children had shorter intensive-care and hospital stays and, in some studies, fewer infections, less respiratory support, and lower reintubation rates [8,17,19,20,23,24]. However, these observational associations do not establish that extubation timing caused the improved outcomes. Children with more favorable baseline conditions, less bleeding, lower vasoactive requirements, better graft function, and uncomplicated abdominal closure are simultaneously more likely to undergo early extubation and recover rapidly. Consequently, shorter ICU or hospital stay cannot be attributed to extubation itself on the basis of the available evidence; selection bias and confounding by indication remain substantial.

This distinction has important implications for the implementation of enhanced-recovery pathways. Immediate extubation should not be treated as an isolated intervention or institutional performance target. Protocol implementation and the formation of dedicated pediatric liver-transplant teams have been associated with increasing extubation rates and fewer postoperative ventilator days, but these improvements probably reflect the cumulative effect of multiple coordinated practices rather than a single anesthetic technique [7,21,24,25]. These practices include predictable anesthetic recovery, opioid-sparing analgesia, quantitative neuromuscular monitoring, goal-directed restoration of perfusion, targeted hemostatic treatment, controlled bleeding, appropriate graft selection and surgical closure, and structured postoperative surveillance. Pharmacological readiness alone is insufficient when hemodynamic or metabolic homeostasis has not been restored.

Institutional context also limits direct comparison among reported extubation rates. Several high rates have been observed in small or selected living-donor-only cohorts [9,11,21]. However, a recent cohort of 99 recipients, 75% of whom received living-related grafts, achieved a 90% immediate-extubation rate within an experienced multidisciplinary program [25], suggesting that high rates are not confined to exclusively living-donor series. These results demonstrate feasibility but should not automatically be generalized to programs with broader recipient severity, different graft availability, greater procedural heterogeneity, or lower transplant volume. The extubation rate itself should not be interpreted as an isolated measure of program quality without adjustment for patient, graft, operative, and institutional characteristics.

Terminological heterogeneity remains another major barrier to interpretation. Combining operating-room extubation with extubation several hours later in the intensive-care unit groups together physiologically distinct events. Similarly, studies define delayed extubation and prolonged ventilation using different thresholds, while reintubation is assessed over variable periods and may include both respiratory failure and reintubation for surgical re-exploration. Future reports should therefore specify the exact location and time of extubation, the duration and type of postextubation respiratory support, the observation window for reintubation, and the reason for reintubation. For comparative purposes, operating-room extubation should be reported separately from early postoperative extubation within 24 h, while delayed and prolonged ventilation should be explicitly defined rather than assumed to be interchangeable.

Available prediction instruments address different stages of the perioperative course. PROVE-ALT estimates prolonged-ventilation risk from pretransplant variables but cannot incorporate intraoperative events [32], whereas proposed end-of-case thresholds based on pH, lactate, urine output, or other physiological measurements remain center-specific and incompletely validated [12,23]. An evidence-informed two-stage framework—baseline risk assessment followed by structured end-of-case reassessment—may be clinically useful, but it requires prospective validation and should not be interpreted as a validated prediction model, score, or universal checklist.

The evidence reviewed has several limitations. The contemporary literature consists almost entirely of retrospective, single-center studies, with substantial variation in recipient selection, graft type, institutional experience, perioperative protocols, extubation definitions, and outcome reporting. Before-and-after pathway studies are additionally susceptible to secular changes and cointerventions. Some perioperative recommendations are extrapolated from adult liver transplantation or broader pediatric critical-care evidence because direct pediatric extubation data remain limited. This narrative review was restricted to English-language publications identified principally through PubMed/MEDLINE and did not include formal risk-of-bias assessment; its conclusions should therefore be interpreted as a clinical synthesis rather than a quantitative estimate of treatment effect.

Future research should prioritize prospective multicenter data collection using standardized definitions and common outcome measures. In addition to extubation timing, studies should report respiratory-failure reintubation separately from procedural reintubation, postextubation respiratory support, airway complications, opioid and sedative exposure, pediatric intensive-care and hospital length of stay, graft and surgical complications, mortality, and resource utilization. Such data would permit external validation of a pediatric two-stage selection framework and clarify whether protocolized immediate or early extubation independently improves outcomes or primarily identifies children who have already achieved successful perioperative recovery.

## 8. Conclusions

Immediate or early extubation can be performed safely in carefully selected pediatric liver-transplant recipients, but baseline characteristics should inform risk rather than serve as absolute exclusion criteria. The decision should emerge from a dynamic end-of-case assessment of respiratory, neurological, neuromuscular, hemodynamic, surgical, and metabolic recovery. Prospective multicenter studies using standardized definitions are needed to validate this evidence-informed framework and determine whether early extubation independently improves outcomes.

## Data Availability

No new data were created or analyzed in this study. Data sharing is not applicable to this article.
